# Overview of the Alberta Kidney Disease Network

**DOI:** 10.1186/1471-2369-10-30

**Published:** 2009-10-19

**Authors:** Brenda R Hemmelgarn, Fiona Clement, Braden J Manns, Scott Klarenbach, Matthew T James, Pietro Ravani, Neesh Pannu, Sofia B Ahmed, Jennifer MacRae, Nairne Scott-Douglas, Kailash Jindal, Robert Quinn, Bruce F Culleton, Natasha Wiebe, Richard Krause, Laurel Thorlacius, Marcello Tonelli

**Affiliations:** 1 Department of Medicine, University of Calgary, Calgary, Alberta, Canada; 2 Department of Community Health Sciences, University of Calgary, Calgary, Alberta, Canada; 3 Department of Medicine, University of Alberta, Edmonton, Alberta, Canada; 4 Calgary Laboratory Services, Calgary, Alberta, Canada; 5 Diagnostic Services of Manitoba, Winnipeg, Manitoba, Canada; 6 Department of Biochemistry, University of Manitoba, Winnipeg, Manitoba, Canada

## Abstract

**Background:**

The Alberta Kidney Disease Network is a collaborative nephrology research organization based on a central repository of laboratory and administrative data from the Canadian province of Alberta.

**Description:**

The laboratory data within the Alberta Kidney Disease Network can be used to define patient populations, such as individuals with chronic kidney disease (using serum creatinine measurements to estimate kidney function) or anemia (using hemoglobin measurements). The administrative data within the Alberta Kidney Disease Network can also be used to define cohorts with common medical conditions such as hypertension and diabetes. Linkage of data sources permits assessment of socio-demographic information, clinical variables including comorbidity, as well as ascertainment of relevant outcomes such as health service encounters and events, the occurrence of new specified clinical outcomes and mortality.

**Conclusion:**

The unique ability to combine laboratory and administrative data for a large geographically defined population provides a rich data source not only for research purposes but for policy development and to guide the delivery of health care. This research model based on computerized laboratory data could serve as a prototype for the study of other chronic conditions.

## Background

The Alberta Kidney Disease Network (AKDN) is a collaborative group of nephrology researchers located in Calgary and Edmonton, Alberta, Canada, with a mission to undertake clinical research and offer research training in kidney disease. The flagship initiative of the AKDN is a unique data collection initiative of routine laboratory tests on all patients in the province of Alberta, resulting in a geographically inclusive database. Patients identified from laboratory data are linked to administrative and other computerized sources to obtain detailed information including socio-demographic data, clinical data including comorbidities, health care encounters, health care costs, death, and kidney-related outcomes.

As the initiative grows the AKDN research findings and activities are beginning to appear more frequently in peer-reviewed literature and health policy circles [1-3]. However, a detailed description of the network's objectives, details of the laboratory database and linked data sources, as well as the process for ongoing patient data collection has not been published. This paper provides such a description, and includes examples of research undertaken as well as future research opportunities for the AKDN.

### Objectives of the AKDN

The AKDN objectives are focused around key research methodologies: clinical epidemiology; health services research; clinical trials; systematic review and meta-analysis; and health economics. Five specific aims relevant to the AKDN dataset were initially identified:

1. To determine the prevalence and identify those at high risk for chronic kidney disease in Alberta, Canada.

2. To determine rates of progression of chronic kidney disease.

3. To determine if access to/quality of specialized medical care and/or rates of progression of kidney disease differs by gender, age, location of residence or ethnic background.

4. To determine the health care costs of caring for patients with chronic kidney disease.

5. To determine optimal treatments for patients with chronic kidney disease.

This is not an exhaustive list, and as the Network grows more objectives and research questions are being addressed. The examples of on-going research projects outlined below demonstrate the potential of this data source.

The AKDN has also developed a plan to facilitate implementation of its research findings. These knowledge translation activities target patients, health care providers, researchers and health policy-makers. These activities aim to provide information to patients regarding kidney function and disease in general, health care providers to assist with investigation and management of patients with chronic kidney disease, and policy makers to guide evaluation and planning of health service delivery for patients with chronic kidney disease. A website http://www.AKDN.info was established to facilitate knowledge translation activities, as well as to provide information regarding team members, training opportunities and research activities.

## Construction and Content

### Laboratory Database Overview

The core component of the AKDN database is the central repository of laboratory data. Through the AKDN, and in collaboration with laboratories across the province of Alberta (population 3.5 million), we have developed a process for retrieval, storage and maintenance of computerized laboratory data and relevant laboratory tests for all patients who have these measurements obtained throughout the province. To permit complete collection of specific tests, and in accordance with privacy laws and ethics board regulations, we were required to limit the number of laboratory tests retrieved and maintained in the database. As such we selected tests which are routinely ordered for patients with common medical conditions to guide ongoing patient assessment, monitor disease progression and identify relevant outcomes (Table 1). The data elements (Table 2) include, in addition to details of the test, a unique patient identifier used for linkage with other data sources. Location of the test (in-versus out-patient) is important to differentiate test results obtained during a hospitalization (potentially influenced by an acute illness), from those obtained in the out-patient setting (which may better reflect stable medical conditions).

**Table 1:** Laboratory tests collected within the AKDN database

Serum Tests:
Creatinine
Hemoglobin
Potassium
Hemoglobin A1C
Fasting total cholesterol
Fasting high density lipoprotein
Fasting low density lipoprotein

**Urine Tests:**

Urine dipstick
Urine microalbumin-creatinine ratio
Urine protein-creatinine ratio
24 hour urine protein

**Table 2:** Data elements in the AKDN laboratory component of the database

Data elements:
Unique patient identifier
Patient date of birth
Patient gender
Test name
Test result
Test date
Health region
Location of test (in- or out-patient)

### Measurement of kidney function

The serum creatinine is used to obtain an estimate of glomerular filtration rate (eGFR). Serum creatinine measurements < 25 umol/L are excluded as they are physiologically implausible. We initially used the non-isotope dilution mass spectrometry traceable 4-variable Modification of Diet in Renal Disease (MDRD) Study equation [4] to estimate GFR, however beginning in 2003 Alberta laboratories began transitioning to methods for creatinine analysis calibrated against an isotope dilution mass spectrometry (IDMS) reference standard. For these creatinine measurements the new version of the MDRD formula derived for use with isotope dilution mass spectrometry traceable creatinine measurements is used [5]. The linear relationship between the old and new methods for estimating GFR was established, thus ensuring accuracy of estimates with these changes in methods over time. To reduce inter-laboratory variation in eGFR creatinine measurements are standardized across provincial laboratories to an IDMS reference standard, and a laboratory-specific correction factor is applied where necessary. Furthermore, we have previously reported minimal intra-laboratory variation of eGFR estimates over time [6]. Although data on race is not available, misclassification of eGFR is expected to be minimal as < 1% of the Alberta population includes individuals of black race [7].

To reduce the effect of regression to the mean, a statistical phenomenon that occurs when repeated measurements with wide variability are made on the same subject [8], baseline kidney function (index eGFR) is estimated using all out-patient serum creatinine measurements taken within a six-month period of the first creatinine measurement, with the index eGFR defined as the mean of the measurements in this six-month period. The date of the last serum creatinine measurement in the six month period, for subjects with more than a single measurement, is used as the index date. Other definitions of baseline kidney function however can be employed, depending on the objectives of the particular study.

Proteinuria, an important predictor of outcomes for patients with kidney disease, is captured by qualitative (urine dipstick) as well as quantitative measures (Table 1). There is considerable variation in guidelines as to the threshold to define clinical significant proteinuria. Our current approach to categorizing urinary protein concentration (Table 3) is based on that proposed by Lamb et al[9].

**Table 3:** Classification of urinary albumin and protein concentration

Classification	Urine albumin:creatinine ratio (mg/mmol)	Urine protein:creatinine ratio (mg/mmol)	24 hour urine microalbumin (mg/day)	24 hour urine protein (mg/day)	Urine dipstick
**Normal**	≤ 2.5 men ≤ 3.5 women	< 15	≤ 30.0	< 150.0	Negative

**Microalbuminuria/Minimal proteinuria**	2.6-29.9 men 3.6-29.9 women	15.0-49.9	31.0-299.9	150.0-299.9	Trace

**Macroalbuminuria/Proteinuria**	30.0-69.9	50.0-99.9	300.0-699.9	300.0-999.9	1^+^

**Heavy macroalbuminuria/Heavy proteinuria**	70.0-315.9	100.0-450.9	700.0-2449.9	1000.0-3499.9	2^+^

**Nephrotic range**	> = 316	≥ 451.0	≥ 2450.0	≥ 3500.0	3^+^

Adapted from Lamb EJ et al [9].

### Linked Data Sources

**Figure 1:**
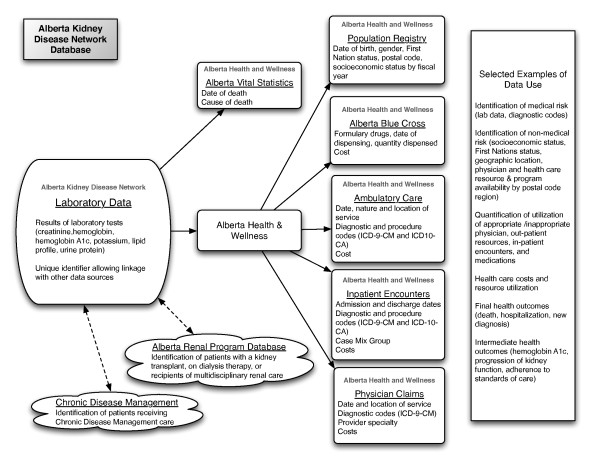
**Data sources and variables linked to the Alberta Kidney Disease Network laboratory data**.

Linkage of laboratory data to administrative and other computerized data sources provides a rich source of information for assessment of socio-demographic characteristics, clinical variables and health outcomes (Figure 1). The unique patient identifier is used to link the laboratory data to a number of such computerized data sources including Alberta Health and Wellness (AHW) administrative data, Alberta Bureau of Vital Statistics, the Northern and Southern Alberta Renal program databases [10], as well as other databases related to program delivery such as the Chronic Disease Management database. All data is available electronically, thus there is no requirement for manual data entry.

AHW administrative data includes all residents of the province of Alberta, and contains data sources which permit determination of disease incidence and prevalence using validated algorithms for common medical conditions such as hypertension, diabetes, acute myocardial infarction, congestive heart failure and stroke [11-15], as well as assessment of clinical, health outcomes and costing information. In addition to routine demographic information and dates of death, the AHW Registry file includes information to permit the assessment of Aboriginal [2], Chinese [16] and South Asian [17] ethnicity, socio-economic status [18], and a six-digit postal code which enables unique geographic information system (GIS) analyses to be performed [19,20].

Alberta Blue Cross administers extended health benefits paid by AHW on behalf of eligible individuals, including residents aged 65 and older, for services which include formulary drugs, permitting evaluation of drug use and its impact on outcomes [21,22]. Details regarding ambulatory care encounters including emergency department visits are obtained from the ambulatory care file, while in-patient hospitalization information (including diagnostic and procedure codes using ICD-9-CM until March 31 2002 followed by ICD-10) is retrieved from the hospitalization file. The physician claims file provides detailed information on all physician encounters, including up to 3 ICD-9 diagnostic codes. The physician claims and hospitalization files are used to define diabetes mellitus and hypertension following validated algorithms [11,12], with other comorbid conditions based on a validated coding algorithm for Charlson comorbidites using ICD-9-CM and ICD-10 codes [23]. The presence of one or more diagnostic code in any position up to 3 years prior to study entry is used for identification of comorbidities.

Follow-up and capture of outcomes of particular relevance for the study of kidney disease, including dialysis initiation or kidney transplantation, is possible by linkage with the Northern and Southern Alberta Renal Program databases [10]. These two programs collectively provide care to all patients with end-stage renal disease in the province. Information on end-stage renal disease status from this clinical database is supplemented by identifying additional long-term dialysis patients from administrative data based on an algorithm for ICD-9-CM codes from physician claims [24].

### Technical Specifications

As outlined in Figure 1, the database consists of linked individual components. The AKDN database operates in a limited access computing environment with a single database server for storage of the data located in Calgary. The AKDN uses the Calgary Health Region server for data storage which maintains security standards required for individual patient level data. Currently the database operates on a Windows operating system. All client computers are personal computers with secure access. All personal identifiers are stripped from the database prior to their use for research purposes to protect patient confidentiality.

### Strengths and Limitations in Use of Laboratory Data for Research Purposes

Laboratory data can be used for case identification and for assessment of disease control, including the use of hemoglobin A1C among patients with diabetes, cholesterol levels among patients at risk for cardiovascular disease, and hemoglobin levels among patients with chronic diseases. It can also be used to identify adverse outcomes related to a treatment or procedure, such as the development of hyperkalemia among patients initiating an angiotensin converting enzyme inhibitor or angiotensin receptor blocker medication. Importantly the serum creatinine measurement can be used to estimate kidney function and define chronic kidney disease, and thus can be used for ongoing surveillance (defined as the ongoing systematic and population-based collection of data).

Surveillance is important for the purposes of disease detection, assessment of trends, identification of service needs for program and policy development, and research. Recently efforts have been directed towards enhanced surveillance for specific chronic medical conditions, including diabetes, cardiovascular disease, and chronic kidney disease. However, the ability to implement a comprehensive surveillance system is limited by the availability of data, and sources currently used often include outcome measures only (such as hospitalization or mortality), or are limited by incomplete assessment of comorbidity. The computerized laboratory data within the AKDN provides the potential for both case identification and ongoing monitoring of chronic medical conditions such as chronic kidney disease (using the serum creatinine to estimate glomerular filtration rate), and can be enhanced to obtain an assessment of comorbidity through linkage with administrative data.

Notwithstanding its strengths, there are important limitations which must be recognized when using the AKDN data. First, the use of laboratory data to define a study cohort by definition will limit the study to subjects who have sought medical care and had a laboratory test undertaken. Although the reasons for measurement are unknown and selected patients may differ from patients without these measurements, this is unlikely to invalidate study findings which are based upon a large proportion of patients from the source population and reflect standard clinical practices in a defined geographic location with universal access to health care. Furthermore a cohort identified by laboratory-based case finding is easily generalized to primary care practice. Second, use of administrative data limits access to certain clinical variables such as blood pressure control and lifestyle factors (smoking, exercise and diet), which may be potential confounders. However other important confounders such as diabetes, hypertension and the Charlson comorbidity index are captured using the computerized data sources.

## Utility and Discussion

The AKDN has used this database to gain a more in-depth understand of chronic kidney disease at the community level, including prevalence and progression of kidney dysfunction [6,25] and aspects of health care delivery for chronic kidney disease [2,3]. Risk factor assessment has also been examined, including the association between drug use and progression of kidney dysfunction [21,22] as well as the impact of anemia on patient outcomes [1]. Other research questions are being explored, including the economic impact of population based screening for chronic kidney disease, the long term outcomes for patients with radio-contrast induced acute kidney injury, and several studies exploring the association between residence location and health outcomes. To date primary users of the database have included both clinician researchers and graduate students within the AKDN. We are currently exploring options for external data requests, which must take into account provincial privacy and security laws.

## Conclusion

The AKDN has developed a unique repository of laboratory data which can be used for health services and health policy research. Although originally developed by nephrology researchers, the AKDN database includes information on all patients who have obtained routine laboratory investigations (not just those with kidney disease), and therefore could also be used for the study of other chronic medical conditions.

## Competing interests

The authors declare that they have no competing interests.

## Authors' contributions

All authors have made substantial contributions to the conception and design of the Alberta Kidney Disease Network (AKDN). All authors have been involved in revising the manuscript for important intellectual content, and all authors have read and approved the final manuscript.

## Pre-publication history

The pre-publication history for this paper can be accessed here:

http://www.biomedcentral.com/1471-2369/10/30/prepub

## References

[B1] Culleton BF Manns BJ Zhang J Tonelli M Klarenbach S Hemmelgarn BR Impact of anemia on hospitalization and mortality in older adults Blood 2006 107 10 3841 3846 10.1182/blood-2005-10-4308 16403909

[B2] Gao S Manns BJ Culleton BF Tonelli M Quan H Crowshoe L Ghali WA Svenson LW Ahmed S Hemmelgarn BR Access to health care among status Aboriginal people with chronic kidney disease CMAJ 2008 179 10 1007 1012 18981441 10.1503/cmaj.080063 PMC2572655

[B3] Hemmelgarn BR Manns BJ Zhang J Tonelli M Klarenbach S Walsh M Culleton BF Association between multidisciplinary care and survival for elderly patients with chronic kidney disease J Am Soc Nephrol 2007 18 3 993 999 10.1681/ASN.2006080860 17267742

[B4] Levey AS Bosch JP Lewis JB Greene T Rogers N Roth D A more accurate method to estimate glomerular filtration rate from serum creatinine: a new prediction equation. Modification of Diet in Renal Disease Study Group Ann Intern Med 1999 130 6 461 470 10075613 10.7326/0003-4819-130-6-199903160-00002

[B5] Levey AS Coresh J Greene T Stevens LA Zhang YL Hendriksen S Kusek JW Van Lente F Using standardized serum creatinine values in the modification of diet in renal disease study equation for estimating glomerular filtration rate Ann Intern Med 2006 145 4 247 254 16908915 10.7326/0003-4819-145-4-200608150-00004

[B6] Hemmelgarn BR Zhang J Manns BJ Tonelli M Larsen E Ghali WA Southern DA McLaughlin K Mortis G Culleton BF Progression of kidney dysfunction in the community-dwelling elderly Kidney Int 2006 69 12 2155 2161 10.1038/sj.ki.5000270 16531986

[B7] Census Canada Census Canada website 2009 http://www12.statcan.ca/english/census06/data/highlights/ethnic/index.cfm?Lang=E 6-15-2009. Ref Type: Electronic Citation 10.1093/ije/dyh299

[B8] Barnett AG Pols JC van der Dobson AJ Regression to the mean: what it is and how to deal with it Int J Epidemiol 2005 34 1 215 220 10.1258/acb.2009.009007 15333621

[B9] Lamb EJ MacKenzie F Stevens PE How should proteinuria be detected and measured? Ann Clin Biochem 2009 46 Pt 3 205 217 10.1258/acb.2009.009007 19389884

[B10] Manns BJ Mortis GP Taub KJ McLaughlin K Donaldson C Ghali WA The Southern Alberta Renal Program database: a prototype for patient management and research initiatives Clin Invest Med 2001 24 4 164 170 11558850

[B11] Quan HKR Hemmelgarn B Tu K Chen G Campbell N Hill MD Ghali WA McAlister F Validation of a case definition to define hypertension using administrative data Hypertension 2009 in press 10.2337/diacare.25.3.512 19858407

[B12] Hux JE Ivis F Flintoft V Bica A Diabetes in Ontario: determination of prevalence and incidence using a validated administrative data algorithm Diabetes Care 2002 25 3 512 516 10.1067/mhj.2002.123839 11874939

[B13] Austin PC Daly PA Tu JV A multicenter study of the coding accuracy of hospital discharge administrative data for patients admitted to cardiac care units in Ontario Am Heart J 2002 144 2 290 296 10.1097/00005650-200502000-00012 12177647

[B14] Lee DS Donovan L Austin PC Gong Y Liu PP Rouleau JL Tu JV Comparison of coding of heart failure and comorbidities in administrative and clinical data for use in outcomes research Med Care 2005 43 2 182 188 10.1161/01.STR.0000174293.17959.a1 15655432

[B15] Kokotailo RA Hill MD Coding of stroke and stroke risk factors using international classification of diseases, revisions 9 and 10 Stroke 2005 36 8 1776 1781 10.1097/01.mlr.0000204010.81331.a9 16020772

[B16] Quan H Wang F Schopflocher D Norris C Galbraith PD Faris P Graham MM Knudtson ML Ghali WA Development and validation of a surname list to define Chinese ethnicity Med Care 2006 44 4 328 333 10.1016/j.puhe.2006.07.001 16565633

[B17] Macfarlane GJ Lunt M Palmer B Afzal C Silman AJ Esmail A Determining aspects of ethnicity amongst persons of South Asian origin: the use of a surname-classification programme (Nam Pehchan) Public Health 2007 121 3 231 236 10.1378/chest.124.1.51 17240412

[B18] Sin DD Svenson LW Cowie RL Man SF Can universal access to health care eliminate health inequities between children of poor and nonpoor families?: A case study of childhood asthma in Alberta Chest 2003 124 1 51 56 10.1378/chest.124.1.51 12853501

[B19] Tonelli M Klarenbach S Manns B Culleton B Hemmelgarn B Bertazzon S Wiebe N Gill JS Residence location and likelihood of kidney transplantation CMAJ 2006 175 5 478 482 16940265 10.1503/cmaj.051356 PMC1550764

[B20] Tonelli M Manns B Culleton B Klarenbach S Hemmelgarn B Wiebe N Gill JS Association between proximity to the attending nephrologist and mortality among patients receiving hemodialysis CMAJ 2007 177 9 1039 1044 10.1038/ki.2008.205 17954893 PMC2025630

[B21] Ahmed SB Culleton BF Tonelli M Klarenbach SW Macrae JM Zhang J Hemmelgarn BR Oral estrogen therapy in postmenopausal women is associated with loss of kidney function Kidney Int 2008 74 3 370 376 10.1016/j.amjmed.2006.02.015 18496507

[B22] Gooch K Culleton BF Manns BJ Zhang J Alfonso H Tonelli M Frank C Klarenbach S Hemmelgarn BR NSAID use and progression of chronic kidney disease Am J Med 2007 120 3 280 e281-287 10.1097/01.mlr.0000182534.19832.83 17349452

[B23] Quan H Sundararajan V Halfon P Fong A Burnand B Luthi JC Saunders LD Beck CA Feasby TE Ghali WA Coding algorithms for defining comorbidities in ICD-9-CM and ICD-10 administrative data Med Care 2005 43 11 1130 1139 10.1097/01.mlr.0000182534.19832.83 16224307

[B24] Oliver M Lok C Shi J Rothwell DM Dialysis therapy for persons with diabetes Diabetes in Ontario: And ICES Practice Atlas 2003 165 180 10.1681/ASN.2007030360

[B25] Gao S Manns BJ Culleton BF Tonelli M Quan H Crowshoe L Ghali WA Svenson LW Hemmelgarn BR Prevalence of chronic kidney disease and survival among aboriginal people J Am Soc Nephrol 2007 18 11 2953 2959 10.1681/ASN.2007030360 17942955

